# In vitro additive effects of dalbavancin and rifampicin against biofilm of *Staphylococcus aureus*

**DOI:** 10.1038/s41598-021-02709-x

**Published:** 2021-12-06

**Authors:** Benjamin Jacob, Oliwia Makarewicz, Anita Hartung, Steffen Brodt, Eric Roehner, Georg Matziolis

**Affiliations:** 1 Orthopaedic Professorship of the University Hospital Jena, Campus Eisenberg, Klosterlausnitzer Straße 81, 07607 Eisenberg, Germany; 2 Institute for Infectious Diseases and Infection Control, Jena University Hospital, 07747 Jena, Germany

**Keywords:** Antimicrobials, Bacteria, Biofilms, Bacterial infection, Musculoskeletal system

## Abstract

Dalbavancin is a novel glycopeptide antibiotic approved for the treatment of acute bacterial skin and skin structure infections (ABSSSIs). It is characterized by a potent activity against numerous Gram-positive pathogens, a long elimination half-life and a favorable safety profile. Most recently, its application for the treatment of periprosthetic joint infections (PJIs) was introduced. The aim of this study was to proof our hypothesis, that dalbavancin shows superior efficacy against staphylococcal biofilms on polyethylene (PE) disk devices compared with vancomycin and additive behavior in combination with rifampicin. *Staphylococcus aureus* biofilms were formed on PE disk devices for 96 h and subsequently treated with dalbavancin, vancomycin, rifampicin and dalbavancin-rifampicin combination at different concentrations. Quantification of antibacterial activity was determined by counting colony forming units (CFU/ml) after sonification of the PE, serial dilution of the bacterial suspension and plating on agar-plates. Biofilms were additionally life/dead-stained and visualized using fluorescence microscopy. Dalbavancin presented superior anti-biofilm activity compared to vancomycin. Additive effects of the combination dalbavancin and rifampicin were registered. Dalbavancin combined with rifampicin presents promising anti-biofilm activity characteristics in vitro*.* Further in vivo studies are necessary to establish recommendations for the general use of dalbavancin in the treatment of PJIs.

## Introduction

Periprosthetic joint infection (PJI) is a persistent problem in orthopedic surgery and treatment remains challenging. The most common pathogens in PJI are coagulase-negative staphylococci (CNS) and *Staphylococcus aureus*^1^. Septic re-revision rates of up to 40.2% within two years after primary arthroplasty were described^2^. Vancomycin is a well-known and established antibiotic used to combat CNS and methicillin-resistant *S. aureus* (MRSA) in PJI^3^. While rifampicin is well known for its biofilm active characteristics^4^, vancomycin shows at least inconsistent activity against biofilm. On the one hand a time-dependend eradication of mature *S. aureus* biofilms could be demonstrated, on the other hand 15 mg/l vancomycin achieved bactericidal activity against only low-biofilm-procuding strains^5,6^. Thus vancomycin susceptibility seems to be heavily strain dependend^7^.

Dalbavancin is a bactericidal lipoglycopeptide antibiotic FDA-approved in 2014 for the treatment of acute bacterial skin and skin-structure infections (ABSSSIs)^8^. Compared to other glycopeptides (e.g. vancomycin), it exhibits a favourable overall safety profile and is characterized by a long elimination half-life, which makes it a suitable candidate for outpatient treatment^9^. Based on these advantages, dalbavancin has been introduced recently as an option for treating PJI^10^. Among the few in vivo studies in rodents that have so far investigated the activity of dalbavancin against biofilms, initial results appear to be promising^11,12^. However, only few studies investigated the synergism and/or additive effects of dalbavancin and rifampicin to date. Therefore, we aimed to determine the efficacy of dalbavancin combined with rifampicin against *S. aureus* biofilms on polyethylene (PE) disks in comparison to both compounds alone and to vancomycin.

## Materials and Methods

### Media and chemicals

Tissue culture dishes (6-well) were obtained from Greiner Bio-One GmbH (Kremsmünster, Austria). Culture media and phosphate buffered saline (PBS) were obtained from Thermo Fisher Scientific/Invitrogen (Karlsruhe, Germany). Antimicrobials were purchased from Merck KGaA (Darmstadt, Germany).

### Biofilm formation

*S. aureus* strain DM 346 was freshly inoculated into CASO Broth (Carl Roth GmbH & Co. KG, Karlsruhe, Germany) and incubated overnight at 35 °C. Thereafter, a bacterial suspension was prepared by dilution the overnight culture 1:100. The polyethylen (PE) disk devices (Mathys AG, Bettlach, Switzerland) were placed in culture dishes and overlayed with 5 ml of the bacteria suspension. Biofilms were grown for 96 h in daily refreshed medium.

### Antimicrobial treatment

PE devices coated by mature biofilms were placed in fresh culture dishes and treated for 48 h with the different antibiotics in concentrations 0, 4, 8, 16, 32 μg/ml, or the dalbavancin/rifampicin combination in concentrations 0, 2, 4, 8, 16 µg/ml of each compound in CASO Broth. Antibiotic-containing medium was refreshed daily.

### CFU determination

After antibiotic treatment, the PE devices were washed with PBS to remove loosely adherent bacteria, and biofilm were detached by sonication for 30 min (BactoSonic, BANDELIN electronic GmbH, Berlin, Germany). The bacteria suspensions were serially diluted to 10^–7^ and 10 μl of dilutions 10^–4^ to 10^–7^ were plated out on Tryptic Soy Agar (TSA) and incubated for 48 h at 35 °C. Colony-forming units (CFU/ml) were determined by counting. Data were obtained from 3 independent experiments, with 3 replicates per condition per experiment.

### Fluorescence microscopy

Biofilms were grown in X-well chamber slides (Sarstedt AG, Nümbrecht, Germany) for 48 h. The supernatant was carefully removed, and the biofilms were treated with the antibiotics as indicated above. After the washing with PBS, biofilms were stained with fluorescein diacetate (FDA, 5 µg/ml) and propidium iodide (PI, 2 μg/ml) for 15 min (Sigma-Aldrich/Merck KGaA, Darmstadt, Germany) and documented by CKX 41 fluorescence microscope (Olympus, Hamburg, Germany).

### Statistical analysis

Statistical analysis was performed using GraphPad Prism Version 5.0.2 (SanDiego, CA, USA) applying Student's paired t-test. A P-value of < 0.05 was considered as statistically significant.

## Results

**Figure 1:**
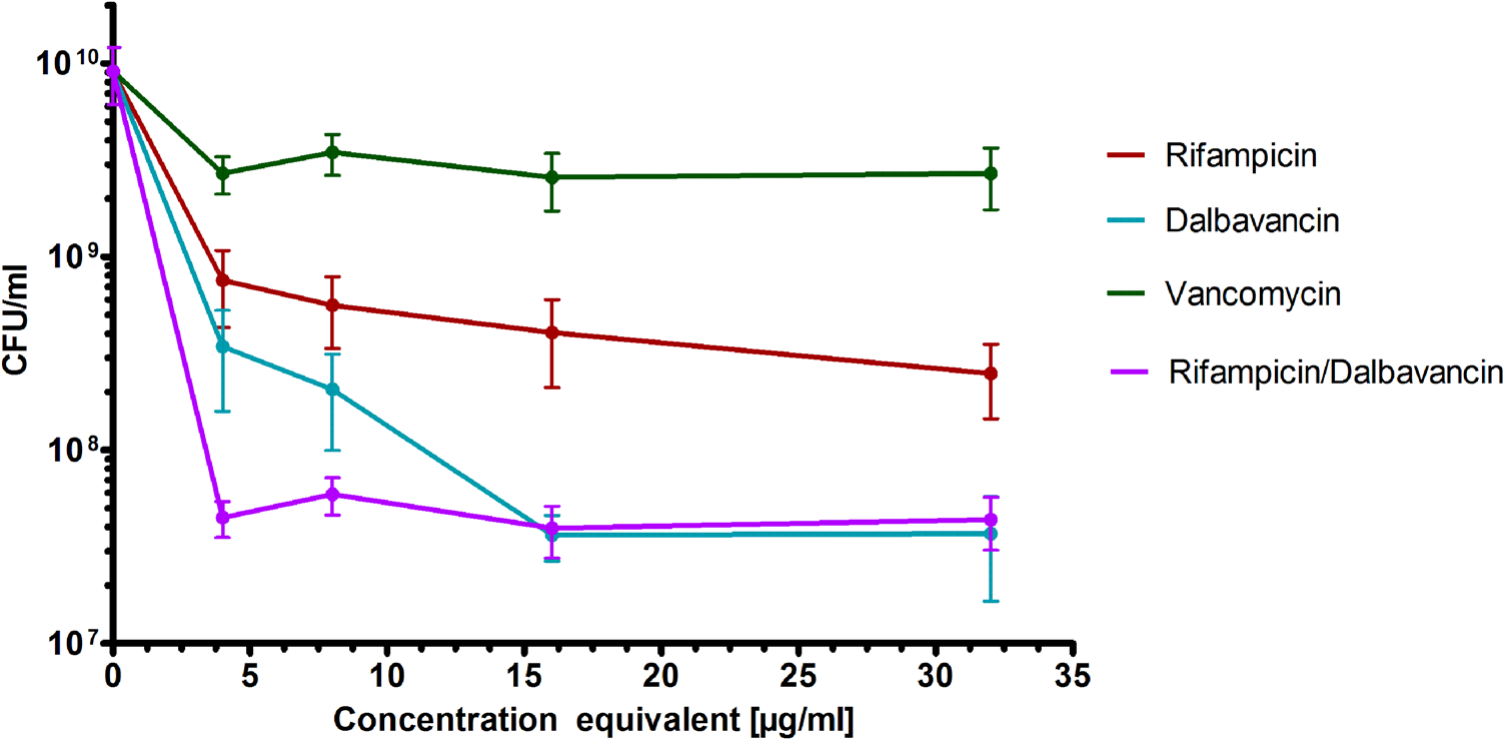
Reduction of CFU/ml of *S. aureus* biofilms grown on polyethylene discs by different antimicrobials. The x-axis represents the concentration equivalents (for details see “Methods” section). Values given as mean ± standard deviation (SD).

The control biofilms grown on the PE discs without antibiotic treatment contained in average (9.1 ± 2.9) × 10^9^ CFU/ml [Fig. 1, intersection with the Y-axis]. Vancomycin, a standard treatment of PJI caused by SA, showed only weak activity against the biofilms reducing the viable bacteria (CFU/ml) by less than one log-magnitude even at its highest concentration. In contrast, dalbavancin and rifampicin exhibited a concentration-dependent anti-biofilm activity with dalbavancin being more effective. At a concentration of 16 mg/l dalbavancin reduced the viable bacteria (CFU/ml) by 2.7 log-magnitudes, while 32 mg/l of rifampicin achieved a reduction of 1.9 log-magnitudes [Fig. 1]. In combination, rifampicin increased the effectiveness of dalbavancin indicating additive effects. The concentration equivalent of the combination at 2 mg/l of each antibiotic was even significantly more effective compared to 4 mg/l of single treatments by dalbavancin or rifampicin. No significant differences could be detected at higher equivalent concentrations [Fig. 1]. In general, the maximum of eradication was achieved by maximum of 3-log-magnitutes (considering single values) by Dalbavancin of Dalbavancin combined with Rifampicin.

**Figure 2:**
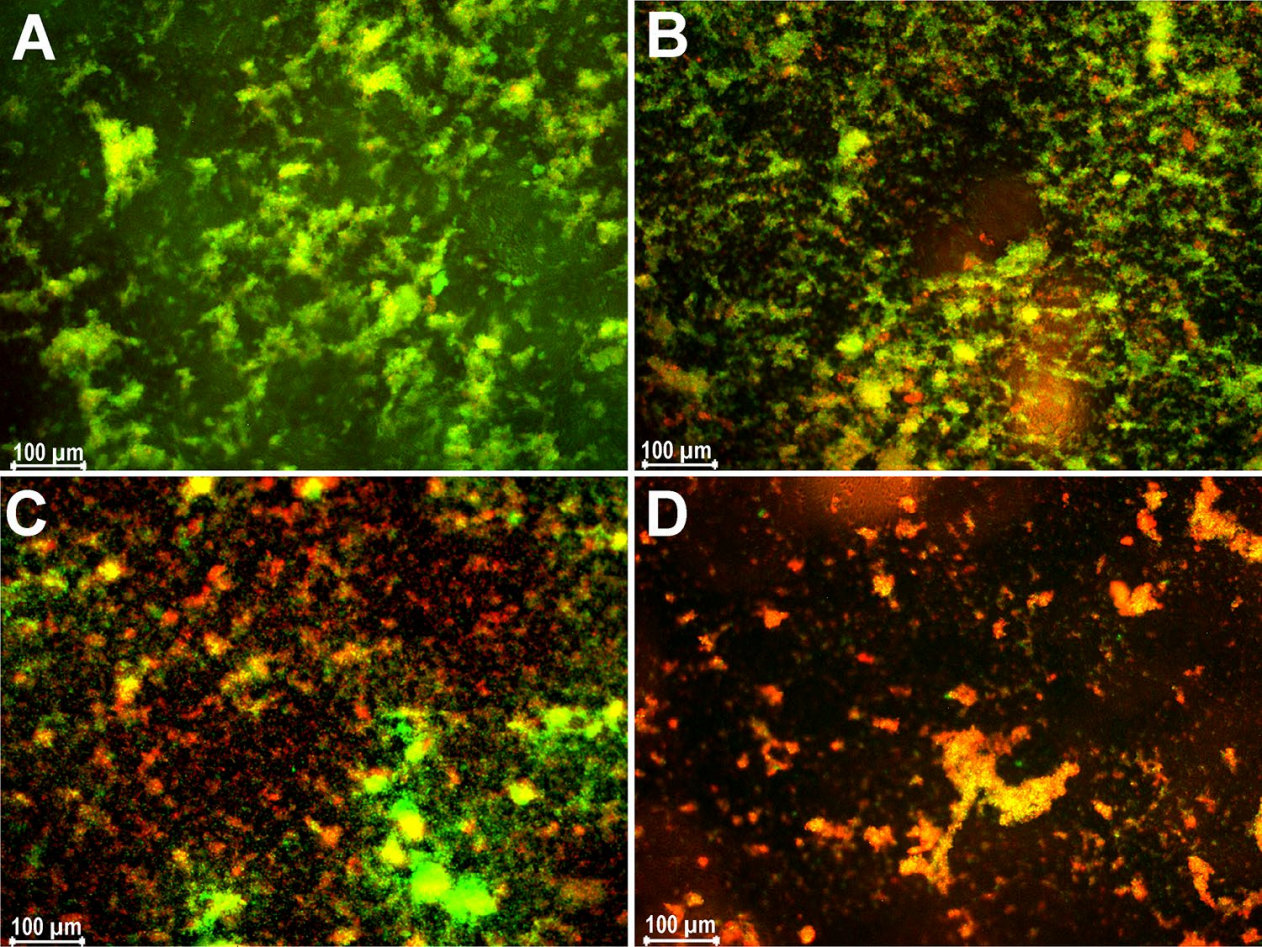
Live/dead stained images of *S. aureus* biofilm treated with (**A**) no antibiotic (control), (**B**) 4 mg/l of dalbavancin, (**C**) 8 mg/l of dalbavancin, (**D**) 32 mg/l of dalbavancin, visualized by fluorescence microscopy. Vital cells stain green (fluorescein positive and PI negative), dead cells stain red (fluorescein negative and PI positive). One representative picture is shown for each treatment.

The microscopic analysis showed a strong reduction of viable cells (green) and emergence of dead cells (red) and an overall destruction of biofilm integrity with increasing dalbavancin concentrations (Fig. 2) supporting the results obtained by the CFU/ml determination.

## Discussion

Biofilm-active antibiotics were shown to be related with better patient outcomes and lower pain intensity in PJI compared to treatment with other antibiotics with weak or no biofilm activity^13^. In the present study, two biofilm active antimicrobials, rifampicin and dalbavancin were thus tested for their synergism and/or additive effects. Dalbavancin is bactericidal and has demonstrated in vitro activity against a wide range of Gram-positive pathogens, including methicillin-resistant *S. aureus* (MRSA)^10^. Dalbavancin binds to the D-Ala-D-Ala terminus of pentapeptide peptidoglycan precursors, thereby inhibiting cell wall peptidoglycan crosslinking^14^. Rifampicin inhibits the translation by interacting with the highly conserved β-subunit of the bacterial RNA-polymerase and thus acts bactericidal on a broad spectrum of bacterial species^15^. As both antimicrobials target different processes in the cell, our hypothesis was that they act synergistically and/or additive against biofilms of *S. aureus*.

The additive effects of dalbavancin and rifampicin could be demonstrated in vitro for *S. aureus* biofilms grown on PE devices. In general, dalbavancin eliminated *S. aureus* biofilms more efficiently than rifampicin, but both antibiotics were superior to vancomycin. The antibiofilm properties of dalbavancin presented in this study correlate with other studies on grafts. In a recent in vitro study, time-kill kinetics of dalbavancin against biofilm of *S. aureus* and *S. epidermidis* grown on cobalt-chrome and titanium disks showed the superiority of dalbavancin over vancomycin^16^. The results can be compared to each other, since bacterial adhesion seems to be similar on different materials (i.e. cobalt-chrome, ceramics, titanium, polyethylene)^17^.

In a recently published in vitro work, the effect of dalbavancin on staphylococcal biofilms when used alone or in combination with biofilm-detaching compounds was examined. It was demonstrated that dalbavancin and rifampicin where the best agents against biofilm formation compared with vancomycin, linezolid and cloxacillin. Regarding established biofilm, the results are consistent with our findings. Although the authors discovered synergistic effects in combination with N-acetylcysteine, direct combination of dalbavancin with rifampicin was not investigated^18^.

There are several limitations of the present study. The experiments were carried out in vitro and cannot fully simulate in vivo conditions. In a foreign-body infection model in guinea pigs, where dalbavancin alone and in combination with rifampicin was used against MRSA, it failed to eradicate the biofilms. The authors concluded that dalbavancin was neither synergistic nor antagonistic with rifampicin, but that it prevented emergence of rifampicin resistance^19^. Thus, the effect in humans might by less synergistic than shown in our present study.

Another bias might come from testing only one *S. aureus* strain as the effect could be strain-specific. However, a study of dalbavancin against biofilms of 171 staphylococci associated with prosthetic joint infections demonstrated high efficacy with minimum biofilm bactericidal concentration MBBC_90_ against *S. aureus* of 2 µg/ml and against *S. epidermidis* of 4 µg/ml, while the MBBC_90_ of vancomycin was > 128 µg/ml. That is far above the achievable concentrations determined with 3.8 µg/ml in cancellous bone or 4.5 µg/ml in cortical bone^20^. This indicates that dalbavancin has a comparable activity in all *S. aureus* sensitive to the antibiotic. However, it should be taken into consideration that both biofilm formation and vancomycin susceptibility vary by strain^6,7^.

Dalbavancin resistance is so far unknown in *S. aureus*, but treatment induced non-susceptibility has been described, e.g. for cardiac device-related endocarditis, mainly due to small colony variant (SCV) formation^21^. As SCV are known to be formed within the biofilms, this might explain, why a reduction of only maximum 3 log-magnitudes could be achieved in our study. Thus, a non-susceptible SCV sub-population might be present in the in vitro biofilms reducing the synergistic and/or additive effect in vivo. SCVs, however were not determined in this study.

Nonetheless, dalbavancin alone and the dalbavancin/rifampicin combination showed promising anti-biofilm activities in vitro*.* The dalbavancin/rifampicin combination was highly effective already at relatively low concentration of 2 µg/ml of each antibiotic. That is even below the expected concentration of both antibiotics in the bone tissue during treatment of PJI. For a rifampicin oral treatment with 600 mg dose twice a day, concentrations were reported of 0.67 ± 0.48 µg/g and 3.35 ± 0.72 µg/g in cortical and spongious bones, respectively^22^. While for a dalbavancin treatment with a single 1000 mg dose, the bone concentration of dalbavancin in cortical bone was 6.3 µg/g after 12 h, and still 4.1 µg/g after 2 weeks^23^. This indicates, that combining both antimicrobials might be a highly effective PJI treatment with only one daptomycin dose and continues rifampicin oral therapy for 14 days. Further in vivo studies are necessary to establish recommendations for the general use of dalbavancin in the treatment of PJIs.
